# Prevention of Acute Radiation-Induced Skin Reaction with NPE® Camellia Sinensis Nonfermentatum Extract in Female Breast Cancer Patients Undergoing Postoperative Radiotherapy: A Single Centre, Prospective, Open-Label Pilot Study

**DOI:** 10.1155/2018/2479274

**Published:** 2018-07-02

**Authors:** Gabriela Näf, Urs E. Gasser, Hans E. Holzgang, Sandra Schafroth, Christoph Oehler, Daniel R. Zwahlen

**Affiliations:** ^1^Department of Radiation Oncology, Kantonsspital Graubünden, Chur, Switzerland; ^2^ClinResearch Ltd., Aesch, Switzerland; ^3^Novelpharm Ltd., Schlieren, Switzerland

## Abstract

**Background:**

To assess effectiveness of NPE, a proprietary Camellia sinensis nonfermentatum (CSNF) extract, in prevention and recovery of acute radiation-induced skin reaction (ARSR) and skin care during postoperative whole breast radiotherapy (RT).

**Methods:**

Twenty patients were enrolled in this single centre, prospective, open-label pilot study. The outcomes of 20 prospective data sets were compared with 100 retrospectively collected matched data sets derived from hospital records. The preventive CSNF gel (2.5%) was administered 1 to 2 hours before each session on the irradiated fields. The care CSNF lotion (0.4%) was administered as 7-day pretreatment after each RT session, twice daily between RT sessions, and 4 to 8 weeks thereafter. The control group was treated according to the hospital care guidelines. The primary endpoint was time to ARSR ≥ Grade 2 (CTCAE v4.03); secondary endpoints were frequencies of ARSR grades 1, 2, 3, and 4, recovery of ARSR, frequencies of interruption and RT stop, complications and required rescue interventions, and tolerability of CSNF.

**Results:**

Time to ARSR ≥ G2 (censoring) was significantly longer (p = 0.014) in the CSNF group. The hazard ratio was 2.33 (95% CI: 1.15–4.72), demonstrating a 50% decrease in the risk of developing ARSR ≥ G2. There was a trend to faster recovery from ARSR G2 in the CSNF group (100% versus 47%; p = 0.078). The proportion of patients requiring rescue treatment during RT and follow-up was markedly higher in the control compared to the CSNF group (1% to 51% versus 0% to 15%). CSNF gel and lotion were well tolerated both during and after RT.

**Conclusions:**

This pilot study provides the first evidence on the potential pharmacological effectiveness of CSNF extract in prevention of RT-induced ARSR and recovery of skin irritation in patients undergoing postoperative whole breast RT and may reflect a novel concept for prevention of RT-induced ARSR and care of irritated skin.

## 1. Background

Breast cancer is the most common female cancer in Switzerland and the leading cause of cancer-related death in European women [1, 2]. For patients with stage I and II disease, breast conserving surgery followed by postoperative radiotherapy (RT) is standard of care. After mastectomy, RT is offered to patients with high risk for relapse, including positive resection margins, involved axillary lymph nodes, and T3-T4 tumors [1]. Acute radiation-induced skin reactions (ARSR) occur in up to 90% of patients undergoing RT, ranging from mild to moderate (Grades I-II) to severe and life-threatening erythema (Grades III-IV) with moist desquamation in over 30% of the patients [3–6]. Development of ARSR may start with the beginning of RT, gradually increase in intensity during RT, and persist for several weeks thereafter [3]. In some patients, toxicity worsens at two to three weeks during RT and may result in more severe ARSR causing pain and impaired quality of life leading to treatment interruption that could potentially reduce the anticancer effectiveness of RT [7].

ARSR is the clinical manifestation of RT interaction with the normal tissue on the molecular and cellular level. Irradiation of cells results in DNA damage by generation of free radicals and reactive oxygen intermediates that cause death of basal epidermal cells, derma, and vascular endothelial cells, therefore triggering inflammatory response inside the tissue [8]. Severity of ARSR is influenced by RT dose, beam energy and fraction size, volume, and technique as well as previous treatments such as type of surgery and chemotherapy regimens [9–12]. ARSR is also affected by patient-related factors including breast diameter and shape, body mass index, and smoking status as well as skin condition at the beginning of RT [11], of which some were recorded in this study.

Currently, there is no established standard of care for prevention and management of ARSR and practices differ between institutions worldwide. Numerous interventions were tested and reported for prevention and management of ARSR; however, it was difficult to conclude any evidence of superiority between these interventions [10, 13]. Only a small number of publications have shown clinically significant results in reducing ARSR including the use of topical corticosteroids [14], hyaluronic acid [15], Calendula officinalis [5], Safetag-based soft silicone (Mepitel) [16], silver leaf dressing [10, 17], and washing with water, with or without mild soap [18, 19].

Previously, skin care products containing green tea plant extract have been studied to treat aging skin caused by ultraviolet radiation [20]. Camellia sinensis nonfermentatum (CSNF) extract used in this study showed reduction of UV-induced erythema, DNA damage, formation of radical oxygen species, and downregulation of numerous factors related to apoptosis, inflammation, differentiation, and carcinogenesis in experimental studies [21]. This generated the hypothesis that similar topical effects could be mediated using CSNF on skin exposed to postoperative RT for breast cancer, as DNA damage/oxidation (direct radiation-induced injury) and oxidative stress (indirect cause of radiation-induced cell injury) are major factors and play an important role in RT-induced normal tissue damage [22, 23]. For treatment prior RT a higher dose gel (aqueous matrix) was used which was not washed off before RT, and a lower dose care lotion (water-in-oil matrix) was used for precare, for skin care after and between RT, and during the subsequent follow-up period. The use of two distinctive CSNF formulations was a consequence of the following facts: Topical administration prior to RT requires an aqueous matrix, e.g., a gel formulation, with a sufficiently high CSNF concentration for a sustained protective (antioxidant) effect against RT-induced skin damage, and fat-/oil-containing formulations must not be used prior to RT as they potentially act as a bolus for flab and increase the risk of RT-induced skin irritations due to a potential dose buildup at the skin surface. On the other hand, for daily skin care and recovery of RT-induced skin damage during the whole RT period, a water-in-oil emulsion matrix with a lower CSNF concentration was considered optimal to achieve the required care effect.

Here we tested the effectiveness of NPE, a proprietary CSNF extract of prevention of ARSR and skin care in this controlled open-label exploratory study conducted in female breast cancer patients undergoing postoperative RT.

## 2. Methods

### 2.1. Ethical Approval and Consent to Participate

This open-label pilot study obtained ethical approval (ClinicalTrials.gov Identifier NCT02500173). Patients included prospectively and patients with retrospective data sets were asked to sign a written informed consent form.

### 2.2. Patients and Treatments

Patients with histologically proven breast cancer who were referred for postoperative whole breast RT were asked to participate in this open-label, prospective monocentric pilot study. Twenty patients were included and received risk-adapted postoperative RT (whole breast: 45 Gy/20 fractions ± boost: 10-15 Gy/4-6 fractions or whole breast: 40 Gy/15 fractions). Patients were instructed to start twice daily administration of the care lotion (0.4% CSNF extract) seven days prior to RT and continue administration during RT on the irradiated fields, except in the morning prior to the next RT session and, if needed, four to eight weeks after completion of RT. The preventive CSNF gel (2.5%) was administered 1 to 2 hours before each RT session on the irradiated fields (breast and supraclavicular front and back region). The 20 prospectively collected data sets of patients treated with CSNF gel and lotion between November 2014 and January 2015 were compared with 100 retrospectively collected data sets derived from hospital records of patients undergoing RT between February 2014 and December 2014. These routine medical care patients were treated according to the hospital treatment guidelines and recommendations of the Scientific Association of Swiss Radiation Oncology (SASRO) (e.g., Excipial® Hydrolotion (oil-in-water emulsion containing urea), Bepanthol® body lotion or Bepanthen® cream (dexpanthenol, a precursor of pantothenic acid or vitamin B5), and Ialugen (sodium hyaluronate) or Ialugen plus cream (sodium hyaluronate and silver sulfadiazine) [24]).

### 2.3. Assessments

The assessments of ARSR Grades 1, 2, 3, and 4 were performed according to the Common Terminology Criteria for Adverse Events (CTCAE) v4.03: dermatitis radiation G1: faint erythema or dry desquamation; G2: moderate to brisk erythema; patchy moist desquamation, mostly confined to skin folds and creases; moderate edema; G3: moist desquamation in areas other than skin folds and creases; bleeding induced by minor trauma or abrasion; G4: life-threatening consequences; skin necrosis or ulceration of full thickness dermis; spontaneous bleeding from involved site; skin graft indicated. ARSR was jointly assessed by a physician and a member of the care team at each RT session (two-rater assessment). The ARSR ≥ G2 was defined as primary endpoint because moist desquamation, even if patchy and often confined to skin folds and creases, represents a clearly defined event and symptom requiring treatment intervention(s). Secondary endpoints were frequencies of ARSR Grades 1, 2, 3, and 4, recovery of skin irritation, defined by reduction of one ARSR grade, frequencies of complications and required interventions (wound infections, smears, and compresses) and rescue treatment (Ialugen cream or Ialugen plus cream), any interruption or stop of RT, and tolerability of preventive gel and care lotion. Assessments were recorded during RT once a week (week 1 to 6) and every two weeks during follow-up (week 2, 4, 6, and up to 8).

### 2.4. Statistical Analysis

The primary efficacy analysis was performed concerning the time from first RT session to diagnosis of ARSR ≥ G2. All occurrences of ARSR ≥ G2 were considered as event. The event date was the first date of diagnosis. Patients without documented event were censored at the date of the last reported visit. Patients who discontinued RT without documented event were censored at the date of discontinuation. If no discontinuation was reported, the last date of visit was defined as censoring date instead. Primary endpoint was analyzed using the stratified Log-Rank tests and presented in a Kaplan Meyer curve.

Secondary quantitative parameters were summarized by descriptive statistics (number of patients, arithmetic mean, and standard deviation, minimum, median, and maximum). Qualitative parameters were summarized by frequency distributions (number of patients, percentages), where n was the actual number of patients with evaluable data for the particular summary statistic frequencies of ARSR Grades 1, 2, 3, and 4 reported in the treatment and standard care group (data with no censoring). Data were analyzed using Fisher's exact test.

## 3. Results

All enrolled patients completed the pilot study according to the protocol, and clinical data were collected prospectively during RT and at follow-up visit(s). Adherence to instructed administration of the CSNF gel and CNSF lotion was checked and revealed compliance in all patients. The comparative control group consisted of 100 patients treated with standard reference care products according the hospital guidelines [24]. Demographics, status, and therapy of breast cancer and RT are presented for the treatment (CSNF gel and lotion) and the control (standard reference care) group in Table 1. Overall demographics, breast cancer therapy, and RT regimens used are comparable between the treatment and control groups. The proportion of patients with mastectomy and chemotherapy were slightly higher in the treatment group, while the proportion of patients with endocrine therapy was slightly lower. The RT (total dose, dose/fraction, dose/boost fraction, and high tangent) was similar in both groups, while the proportion of patients with hypofractionation and nodal irradiation was slightly lower in the CSNF group. At our institution, hypofractionation was introduced as standard of care in patients over 60 years of age or for patients with endocrine responsive and node negative breast cancer not requiring a boost within the tumor bed. The individually required follow-up periods varied according to skin conditions after the last RT session and individual requirements for subsequent skin care, follow-up interventions, and rescue treatments (Ialugen cream, Ialugen plus cream).

**Figure 1:**
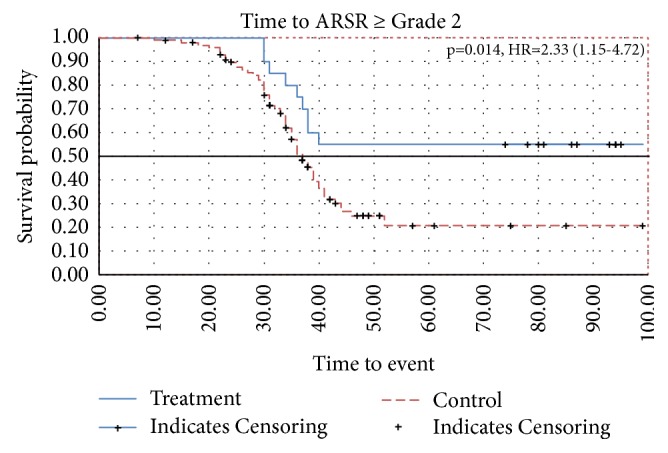
Time to ARSR ≥ Grade 2. p=0.014, HR=2.33 (1.15-4.72). Censoring: patients without documented event will be censored at the date of the last reported visit date. Patients having discontinued the radiotherapy without documented event will be censored at the date of discontinuation. If no discontinuation date is reported the visit date will be taken as censoring date instead. x-axis: months; y-axis: probability.

**Figure 2:**
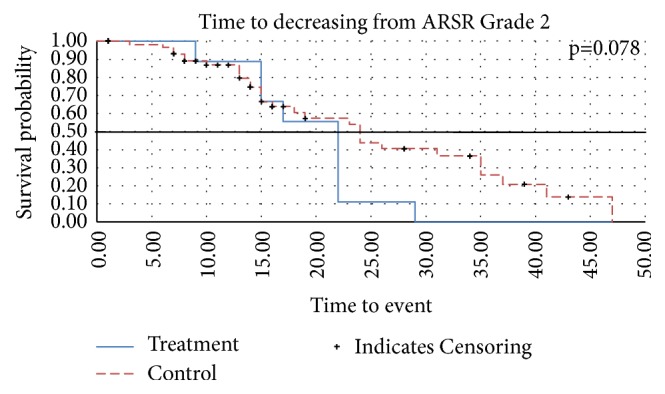
Time to recovery from ARSR Grade 2. p=0.078. Censoring: patients without documented event will be censored at the date of the last reported visit date. Patients having discontinued the radiotherapy without documented event will be censored at the date of discontinuation. If no discontinuation date is reported, the visit date will be taken as censoring date instead. x-axis: months; y-axis: probability.

Time to ARSR ≥ G2 is presented in a Kaplan Meier curve in Figure 1 (censored data). In the treatment group the time to ARSR ≥ G2 was longer (p = 0.014), hazard ratio (HR) = 2.33 (95% confidence interval (CI): 1.15–4.72). The proportion of patients experiencing ARSR ≥ G2 (no censoring) was 45% in the treatment group and 62% in the control group (p=0.213), and the mean (SD) time was 34.9 (3.8) and 32.6 (7.8) days, respectively (Table 2). The time of skin recovery, defined by decrease of ARSR ≥ G2 by one ARSR grade, is presented in a Kaplan Meier curve in Figure 2 and showed a trend in favor for the CSNF, but failed to be significant (p=0.078). Skin recovery was checked and recorded at each visit in the prospective cohort and derived from the hospital records of the retrospective cohort.

**Figure 3:**
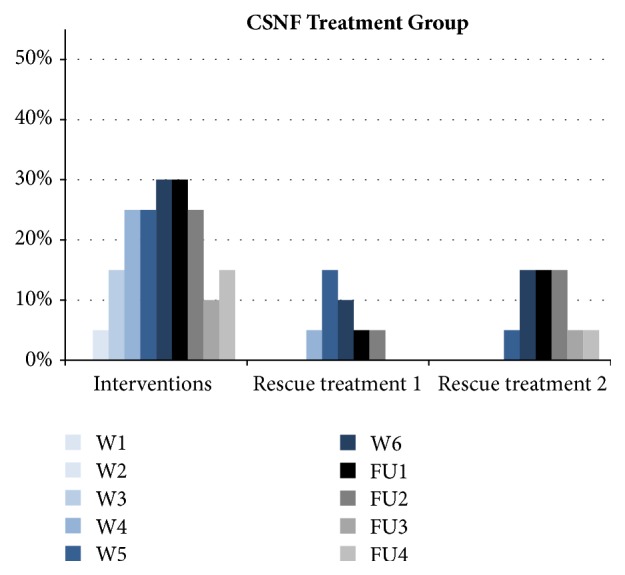
Interventions (wound smears and compresses) and rescue treatment 1 (Ialugen® cream) and rescue treatment 2 (Ialugen plus cream) in the CSNF treatment group. The denominator is count of patients in the regarding population (CSNF group: n=20; standard reference treatment group: n=100). W: week; FU: follow-up.

**Figure 4:**
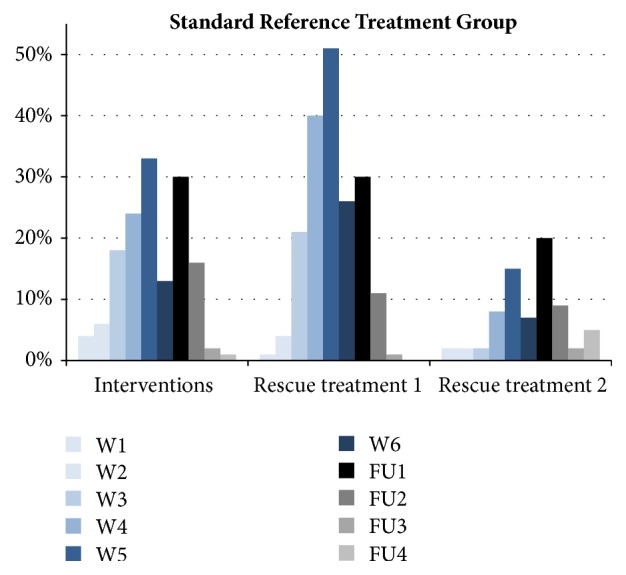
Interventions (wound smears and compresses) and rescue treatment 1 (Ialugen cream) and rescue treatment 2 (Ialugen plus cream) in the standard care reference treatment group. The denominator is count of patients in the regarding population (CSNF group: n=20; standard reference treatment group: n=100). W: week; FU: follow-up.

Time to ARSR Grades 1, 2, and 3 was not significantly different when comparing the CSNF and control group using the exact Fisher test (no censoring). ARSR G4 was not reported in any patient (Table 2). Patients requiring interventions such as compresses and wound smears (to check for potential infections) were recorded as of week 2 in the CSNF group and as of week 1 in the control group. Incidences of required interventions rose up to a maximum of 30% in the CSNF group at RT week 6 and follow-up week 2 and up to a maximum of 33% in the control group at RT week 5 (Figures 3 and 4). Rescue treatments 1 (Ialugen cream) and 2 (Ialugen plus cream) were required in the control group as of week 1 and in the CSNF group as of weeks 4 and 5, respectively. The proportion of patients requiring rescue treatment 1 reached a maximum of 15% in the CSNF group and 51% in the control group, and the proportion of patients requiring rescue treatment 2 reached a maximum of 15% in the CSNF group and 20% in the control group (Figures 3 and 4).

## 4. Discussion

This is the first study using a combination of a CSNF gel and lotion preventing and rescuing ARSR in patients with breast cancer undergoing postoperative whole breast RT. The most important findings of this trial were the significant delay of ARSR grade ≥ 2 events (primary endpoint, p = 0.014) and a trend in decreasing the overall risk of ARSR grade ≥ 2 events by about 50%. The combination of a CSNF gel (2.5%) administered prior to each RT session and a CSNF lotion (0.4%) before, during, and after the RT was well tolerated.

In the control group, we used the standard care treatments for ARSR recommended by the SASRO nursing group [24] similarly to many other Swiss RT departments. Excipial Hydrolotion and Bepanthol body lotion or Bepanthen cream were applied on patients with ARSR G1 and G2, whereas for patients with moist desquamation (ARSR ≥ G2), Ialugen was administered or Ialugen plus (antimicrobial effect); if there was evidence for an infected wound after tissue, swap was taken for diagnostic purposes. Both dexpanthenol and sodium hyaluronate creams showed efficacy in preventing ARSR > G2 in clinical trials [15, 25, 26]. However, there were limitations in these trials such as small patient samples or inclusion of a heterogeneous study population, and patient-reported outcomes were not communicated [13]. As a consequence, neither the use of dexpanthenol nor the use of sodium hyaluronate cream could be considered standard treatment for ARSR.

We compared the outcomes of the prospective pilot study in 20 patients treated with the CSNF preventive gel and lotion with the SASRO standard care treatment of 100 women of the retrospective cohort. Despite the difference in patient number included, the two groups were reasonably well balanced with respect to age, surgical procedures, endocrine therapy, and chemotherapy. In terms of RT, total dose, fractions size, and boost application were similar in both groups. The use of hypofractionation and nodal irradiation was recorded at a higher percentage in the standard group (Table 1), and no increase of ARSR ≥ G2 was recorded in either group. This finding is in accordance with the current literature on the favorable use of hypofractionation in reducing the frequency of ARSR ≥ G2 in patients with breast cancer undergoing whole breast RT [9].

This pilot study demonstrated that the combined use of CSNF gel and lotion delayed the occurrence of ARSR ≥ G2 and may reduce the risk of moist desquamation of the irradiated skin by 50% [HR 2.33 (95% CI: 1.15 - 4.72)]. Also, the requirement of rescue treatments 1 (Ialugen cream) and 2 (Ialugen plus cream) was delayed in the CSNF group, and the proportion of patients requiring rescue treatments was lower in the CSNF group (Figure 3). The CSNF extract reduces oxidative stress and DNA damage, downregulates numerous factors related to apoptosis, inflammation, and carcinogenesis in experimental studies, and showed a similar protective effect for skin of healthy volunteers exposed to UV-light [21]. As CSNF has not been tested before in postoperative whole breast RT in patients with breast cancer, a direct comparison of our data with other published results is not possible.

Several studies analyzing prophylaxis of radiation-induced side effects of patients undergoing postoperative RT showed a similar or even higher magnitude of risk reduction of ARSR ≥ G2 [17]. The randomized study by Herst et al. including 80 patients and using Mepitel Film, a Safetac technology-based soft silicone dressing, in a prophylactic manner, demonstrated complete prevention of ARSR ≥ G2 according to the RTOG scale and a 92% reduction in overall skin reaction severity. As the RTOG scale is comparable to the CTCAE 4.03 system, this finding highlights the fact that maintaining the integrity of the irradiated skin with either mechanical protection or pharmacological properties of an extract, such as CSNF, may prevent damage of the basal skin layer [16].

The second important observation was the improved recovery of ARSR ≥ G2 compared to the control group. Although this result was not statistically significant, most likely due to the small number of patients included, it is another argument why the use of rescue treatment with Ialugen plus cream (sodium hyaluronate and silver sulfadiazine) was less frequent in the CSNF group (Figure 3). A rapid recovery of ARSR ≥ G2 events not only would improve quality of life of patients during and after postoperative RT but also could have an impact on costs of care.

When assessing and grading ARSR in our prospective patient cohort, we noted some interobserver variability despite the fact that ARSR appears to be describable in an objective and reproducible manner using the standardized CTCAE v4.03 scoring system. Neben-Wittich et al. demonstrated in their work assessing 166 patients with ARSR that interobserver variability among radiation oncologists was much higher when measuring subjective symptoms such as pain, itching, burning, or irritation of the skin in the RT field. The reason was that CTCAE grade of ARSR did not correlate well with the patients' symptom experience applying patient-reported outcome measures [27]. This is of importance as patients in our study asked for tailor-made management for their ARSR presenting with multiple symptoms (e.g., itching and burning) not worrying about the performed ARSR grading by the responsible physician.

Absence or inconsistencies of patient-reported outcomes are therefore a major obstacle in clinical studies aiming to improve ARSR in patients undergoing postoperative RT for breast cancer. Correct evaluation of their intensity is very important as this helps to predict the probability of consequential late side effects in this patient group [4]. Patient-reported outcome measures should therefore be included besides provider-reported toxicity assessments in the design of a clinical trial assessing ARSR of patients undergoing postoperative RT for breast cancer. They serve as a tool for an adequate, prognostic, and standardized assessment of ARSR [27, 28].

Our study may also include some limitations. First, this was a pilot study with a small number of patients included. The prospective cohort included 20 patients, and the control group consisted of 100 patients, and its retrospective nature introduced a selection and reporting bias. Also, the CSNF gel and lotion were used as “open-label” drugs, and there was no “blinded” placebo control. Second, the assessment of ARSR was based on ratings by the treating physician and nursing staff, and patient-related outcome measures were not integrated. Third, no standardized photodocumentation was performed to track and compensate for potential interobserver variability, despite two-rater assessment strategy. Fourth, the secondary study endpoint “time to recovery” was evaluated also in the follow-up visits and this most likely introduced a lead-time bias. Finally, there was a higher and earlier requirement for rescue treatments 1 (Ialugen cream) and 2 (Ialugen plus cream) in the control group. The lower and delayed need of rescue treatments in the CSNF group may indicate also a preventive effect of the CSNF pretreatment before the start of the RT.

Further investigations on a larger group in a randomized, parallel, placebo-controlled design are required in order to substantiate and confirm outcomes derived from this pilot study. There is a medical need for developing a novel concept for prevention of RT-induced ARSR and care of irritated skin, and patients would benefit by improvement of quality of life during and after RT.

## 5. Conclusion

This study conducted in routine medical care provides the first evidence that this Camellia sinensis nonfermentatum extract is potentially effective in prevention of RT-induced ARSR and recovery of skin irritation in females with breast cancer undergoing postoperative RT and may reflect a novel concept for prevention of RT-induced ARSR and care of irritated skin.

## Figures and Tables

**Table 1:** Demographics, status, and therapy of breast cancer and radiotherapy.

	CSNF Treatment group (n=20)	Standard care group (n=100)
Age (years)	60.2 (11.9)	62.7 (12.4)

Weight (kg)	70.5 (14.4)	75.8 (14.8)

Type of skin		Not recorded

II-Nordic Type	35%	

III-Mixed Type	40%	

IV-Mediterranean Type	25%	

Breast size		Not recorded

B	55%	

C	35%	

D	10%	

Breast cancer therapy		

Breast conservation	90%	90%

Mastectomy	9%	5%

Endocrine Therapy	48%	55%

Chemotherapy	38%	25%

Radiotherapy		

RT total dose (Gy)	55.4 (5.9)	54.8 (7.6)

Planned number of fractions	23.9 (3.8)	23.4 (4.4)

Dose/fraction (Gy)	2.3 (0.2)	2.4 (0.2)

Number of boost fractions	5.6 (0.8)	5.3 (1.4)

Dose/Boost fraction (Gy)	2.4 (0.2)	2.5 (0.1)

Hypo-fractionation	15%	25%

High tangential fields	11%	10%

Nodal irradiation	14%	30%

Mean (SD); percentage (%).

**Table 2:** Frequency of ARSR grades and time to ARSR Grades 1, 2, 3, and 4.

	Treatment group: CSNF gel and lotion	Standard care reference group	p-value^*∗*^
ARSR Grade 1 Frequency	95%	92%	ns

Time to (days)	20.1 (12.4)	22.7 (8.5)	
	23 (1-38)	22 (1 -51)	

ARSR Grade 2 Frequency	45%	62%	p=0.213

Time to (days)	34.9 (3.8)	32.6 (7.8)	
	36 (30-40)	34 (10-52)	

ARSR Grade 3 Frequency	15%	9%	ns

Time to (days)	39.7 (5.5)	44.1 (5.7)	
	37 (36-46)	43 (36-56)	

ARSR Grade 4 Frequency	0%	0%	NA

## Data Availability

Data are available on request.

## Abbreviations

ARSR: Acute radiation-induced skin reaction
CSNF: Camellia sinensis
CTCAE: Common terminology criteria of adverse events
G: Grade
RT: Radiotherapy
RTOG: Radiation Therapy Oncology Group
SASRO: Scientific Association of Swiss Radiation Oncology
UV: Ultra violet
HR: Hazard ratio
CI: Confidence interval
SD: Standard deviation.
